# The mRNA COVID-19 vaccine in patients with cancer receiving checkpoint inhibitor therapy: what we know and what we don't

**DOI:** 10.2217/imt-2021-0235

**Published:** 2021-11-08

**Authors:** Alexandre E Malek, Paulette Pinargote Cornejo, Nour Daoud, Mohammad Alam

**Affiliations:** ^1^Department of Medicine, Division of Infectious Diseases, Louisiana State University Health Sciences Center, Shreveport, LA 71103, USA

**Keywords:** booster, cancer, checkpoint inhibitors, immunotherapy, mRNA COVID-19 vaccine

The COVID-19 pandemic has led to heroic achievements in vaccine development targeting the spike protein of SARS-CoV-2. Importantly, the vaccine produces neutralizing antibodies (Abs) and induces a T-cell response against the spike protein; however, there are no approved tests to assess the T-cell-mediated immune and protective response against COVID-19 and there is no correlation between the antibody level and protection against the virus. During the early COVID-19 vaccine trials, patients with cancer were generally excluded from the studied populations [1]. Nonetheless, given the increased mortality rate of up to 13% of patients with cancer and COVID-19 infection and the worrisome complications including delays in cancer treatment, the National Comprehensive Cancer Network and other oncologic societies, such as the American Society of Clinical Oncology and European Society for Medical Oncology, have recommended the COVID-19 vaccine to patients with active cancer regardless of therapy [2–4]. Multiple cohorts have now been examined, and a recent study evaluating the efficiency of the mRNA COVID-19 vaccine in patients with hematological malignancies found that patients who were actively receiving BTKIs, ruxolitinib, venetoclax or anti-CD20 antibody therapies mounted a blunt vaccine-immune response, were unprotected from the SARS-CoV-2 infection and could still develop severe and critical disease [5]. Similar studies show the reduced mRNA vaccine immunogenicity in patients receiving immunosuppressive medications, such as rituximab or mycophenolate, and in patients with liquid tumors [6].

## How checkpoint inhibitors can interfere with COVID-19 vaccination

A subset of patients with cancer who receive checkpoint inhibitor (CPI) therapy may have a unique response to the COVID-19 vaccine, since CPIs enhance T-cell activity, prevent immune exhaustion and may boost vaccination response [7]. Extrapolating from influenza vaccine studies, despite the lack of robust prospective clinical data, showed that patients with cancer who received CPIs had a stronger and adequate T-cell response compared with those who were treated with cytotoxic chemotherapy [8,9]. In contrast to the effects of CPIs, Shroff *et al.* showed that COVID-19 vaccine-induced antibody and T-cell response was substantially reduced in patients with solid tumors receiving active cytotoxic chemotherapy compared with individuals not on immunosuppressive therapy [10]. A more recent prospective cohort study reported on the immune response of the BNT162b2 mRNA COVID-19 vaccine in 102 patients with cancer with solid tumors who were actively receiving chemotherapy compared with 78 healthy patients. In this study, 90% of patients with cancer had adequate antispike IgG antibody response after a median of approximately 5.5 weeks following the second vaccine dose, compared with 100% of patients in the control group, although their Abs titers were lower than those of healthy controls [11]. Out of 102 patients, 22 received immunotherapy as monotherapy. Interestingly, those who received CPIs alone were among the patients with the highest SARS-CoV-2 antispike IgG titer values [11]. In a subsequent report from the same study, patients were followed up to 4 months after the second vaccine dose, and the seropositivity rate to antispike IgG Abs was 87% in the cancer group and remained 100% in the control group. Similar to the early analysis at 5.5 weeks, the highest IgG titers were mainly seen in patients receiving either immunotherapy alone or biological therapy, in contrast to those who received a combination of chemotherapy plus immunotherapy [12]. A prospective clinical trial (VOICE) is underway, with the primary end point being the assessment of the antibody response at day 28 following the second dose of the COVID-19 vaccine, and is enrolling patients with cancer treated with chemotherapy, immunotherapy or chemoimmunotherapy [13].

While the use of CPIs may enhance the vaccine immunogenicity, it is not uncommon that patients receiving CPIs may develop immune-related adverse events (irAEs) that will require treatment with high doses of corticosteroids or other immunosuppressant drugs that could affect the patient's ability to mount a protective immune response. Deepak *et al.* studied mRNA COVID-19 vaccine-induced immunogenicity in 133 adults with chronic inflammatory diseases (CIDs) versus 53 immunocompetent controls and found that patients treated with immunosuppressive therapies display a significant reduction in antispike IgG titers, primarily in those treated with corticosteroids (seropositivity postvaccination decreased to 65% in patients on prednisone) and B-cell depletion therapy, while exposure to TNF-α inhibitors, IL-12/23 inhibitors and integrin inhibitors had only a minimal impact on the humoral response and antibody neutralization function [14].

## How COVID-19 vaccines can impact checkpoint inhibitor immunotherapy

Medical experts have raised concerns regarding the safety and ability of the vaccine to provoke enhanced irAEs in patients treated with CPIs; however, Waissengrin *et al.* showed the safety of mRNA vaccine in this patient group without observing any vaccine- or CPI-related side effects following the second vaccine dose [15]. In addition, Chen *et al.* conducted another retrospective study by reviewing the medical records of patients with cancer who received CPIs within one month of receiving the COVID-19 mRNA vaccine and were followed for at least one month after the second dose of vaccine. The researchers also reviewed data from the US FDA's Vaccine Adverse Event Reporting System (VAERS) for possible irAEs following COVID-19 vaccine administration. Notably, there was no indication of increased risk of new or worsening pre-existing irAEs following vaccine administration, which further supports the safety of the COVID-19 vaccine in patients with cancer receiving CPIs [16]. On the other hand, it has been reported that certain patients with solid tumors receiving CPIs may have a paradoxical response consisting of tumor hyperprogression, and it remains unknown whether vaccines can enhance this unexpected phenomenon in this patient subgroup [17].

## Areas of uncertainty

One layer of host immune response against COVID-19 is the mucosal-associated invariant T (MAIT) cells, which are innate T-cell subsets involved in mucosal immunity and viral clearance and can play the role of hero, villain or both in COVID-19 infection [18]. Flament *et al.* demonstrated that MAIT cells can have double-edged effects in displaying a cytotoxic profile in the lungs and contributing to deleterious inflammation, vital organ damage and severe COVID-19 infection [19]. Another study showed that alteration in MAIT cells' function may trigger an uncontrolled immune response leading to further tissue damage in COVID-19 [20]. On the other hand, researchers found that MAIT cells play a role in adenovirus vector vaccines, which mediate the T-cell response in humans and may serve as an additional pathway to achieve optimal vaccine immunogenicity [21]. The effects of MAIT cells could be exploited for a better understanding of the immunologic response to the mRNA vaccine. Of interest, a prospective study that evaluated patients with melanoma who were treated with CPIs and had COVID-19 infection found that CPI therapy was not associated with severe COVID-19 disease or exacerbated inflammation, but led to an increased and amplified antiviral T-cell immune response. These findings warrant further exploration to help enhance vaccine efficacy. In addition, more prospective studies are needed to characterize the exact impact of CPIs on SARS-CoV-2-infected patients [22,23]. In contrast to the potentially harmful role of MAIT cells and the role of CPIs in SARS-CoV-2-infected patients, it has been anecdotally found that circulating MAIT cells in the blood were higher in patients with cancer who were responding to immunotherapy [24].

## COVID-19 vaccine booster

In light of the growing number of reported breakthrough COVID-19 infections among fully vaccinated immunocompromised hosts and the emerging data suggesting that a third booster vaccine dose enhances the immune antibody response in solid-organ transplant recipients and hemodialysis patients, the FDA and CDC promptly issued a recommendation and approved the administration of an additional dose of mRNA vaccine for moderately to severely immunosuppressed patients to be given at least 28 days after the completion of the initial two-dose mRNA COVID-19 vaccine series [6,25]. Hence, patients with cancer, including solid tumors and hematologic malignancies receiving active anticancer therapies including CPIs, are the most vulnerable and at risk for severe COVID-19 and related mortality, and would therefore benefit from an additional COVID-19 vaccine dose.

## Conclusion

A third dose of the vaccine may substantially benefit those with cancer who have weak antibody and T-cell responses. Further detailed analyses assessing the function of T-cell subsets following vaccination will be required to understand the effect of CPIs on the immune response. That said, patients with cancer show vast heterogeneity in terms of cancer types, anticancer therapies and management of irAEs; therefore, it is difficult to individualize vaccination on a case-by-case basis. Given the dramatic impact of COVID-19 on patients with cancer, and while more studies at the granular level are needed, the COVID-19 vaccination is no doubt the most effective preventive measure. The main concern remains that combined CPI therapy (anti-PD-1, anti-PD-L1 and anti-CTLA-4) may potentiate the risk of irAEs regardless of the vaccine. These patients should be closely monitored during the COVID-19 vaccination process.

## Future perspective

Many questions remain to be addressed for patients receiving CPIs regarding the timing of the COVID-19 booster vaccination and the likelihood of an increase in irAEs following the third dose. A clinical trial assessing the safety and immunogenicity of the third mRNA COVID-19 vaccine dose in patients with solid or lymphoid tumors on CPI immunotherapy would be informative and timely. Theoretically, patients who respond to CPI immunotherapy may have a stronger immune response following the COVID-19 mRNA vaccine, given the increased circulating MAIT cells in the blood, which could serve as an additional pathway for enhancing vaccine immunogenicity. Should we prioritize the nonresponders to CPIs to receive the third dose of vaccine? An improved understanding of the specific role of MAIT cells in mRNA vaccine response is needed. Considering the high mortality rate from COVID-19 among patients with cancer, the benefit of a booster vaccine dose exceeds the hypothetical increased risk of irAEs. In addition, CPIs boost T-cell functions and may potentiate T cell-response and, subsequently, humoral-response to the COVID-19 vaccine and induce long-term immunity; however, this remains to be investigated.

## References

[1] Corti C, Curigliano G. Commentary: SARS-CoV-2 vaccines and cancer patients. Ann. Oncol. 32(4), 569–571 (2021). 33450404 10.1016/j.annonc.2020.12.019 PMC7831848

[2] The National Comprehensive Cancer Network. NCCN: Cancer and COVID-19 vaccination. (2021) www.nccn.org/docs/default-source/covid-19/2021_covid-19_vaccination_guidance_v3-0.pdf?sfvrsn=b483da2b_60. 10.6004/jnccn.2021.5100 34818626

[3] American Society of Clinical Oncology. COVID-19 vaccines & patients with cancer. (2021). www.asco.org/asco-coronavirus-information/covid-19-vaccines-patients-cancer.

[4] European Society for Medical Oncology. COVID-19 vaccination in cancer patients: ESMO statements. (2021) www.esmo.org/covid-19-and-cancer/covid-19-vaccination.

[5] Maneikis K, Šablauskas K, Ringelevičiūtė U Immunogenicity of the BNT162b2 COVID-19 mRNA vaccine and early clinical outcomes in patients with haematological malignancies in Lithuania: a national prospective cohort study. Lancet Haematol. 8, e583–e592 (2021). 34224668 10.1016/S2352-3026(21)00169-1 PMC8253543

[6] Oliver S. Data and clinical considerations for additional doses in immunocompromised people. Presented at: ACIP Meeting. 22 July 2021.www.cdc.gov/vaccines/acip/meetings/downloads/slides-2021-07/07-COVID-Oliver-508.pdf.

[7] Korompoki E, Gavriatopoulou M, Kontoyiannis DP. COVID-19 vaccines in patients with cancer-a welcome addition, but there is need for optimization. JAMA Oncol. 7(8), 1114–1114 (2021). 10.1001/jamaoncol.2021.1218 33983372

[8] Kang CK, Kim H-R, Song K-H Cell-mediated immunogenicity of influenza vaccination in patients with cancer receiving immune checkpoint inhibitors. J. Infect. Dis. 222(11), 1902–1909 (2020). 32479600 10.1093/infdis/jiaa291

[9] Bersanelli M, Buti S, de Giorgi U State of the art about influenza vaccination for advanced cancer patients receiving immune checkpoint inhibitors: when common sense is not enough. Crit. Rev. Oncol. Hematol. 139, 87–90 (2019). 31112887 10.1016/j.critrevonc.2019.05.003

[10] Shroff RT, Chalasani P, Wei R Immune responses to two and three doses of the BNT162b2 mRNA vaccine in adults with solid tumors. Nat. Med. (2021) (Epub ahead of print). 10.1038/s41591-021-01542-z PMC9004706 34594036

[11] Massarweh A, Eliakim-Raz N, Stemmer A Evaluation of seropositivity following BNT162b2 messenger RNA vaccination for SARS-CoV-2 in patients undergoing treatment for cancer. JAMA Oncol. 7(8), 1133–1140 (2021). 34047765 10.1001/jamaoncol.2021.2155 PMC8164144

[12] Eliakim-Raz N, Massarweh A, Stemmer A, Stemmer SM. Durability of response to SARS-CoV-2 BNT162b2 vaccination in patients on active anticancer treatment. JAMA Oncol. e214390 (2021). 10.1001/jamaoncol.2021.4390 PMC8358809 34379092

[13] van der Veldt AAM, Oosting SF, Dingemans AMC COVID-19 vaccination: the VOICE for patients with cancer. Nat. Med. 27(4), 568–569 (2021). 33589821 10.1038/s41591-021-01240-w

[14] Deepak P, Kim W, Paley MA Effect of immunosuppression on the immunogenicity of mRNA vaccines to SARS-CoV-2: a prospective cohort study. Ann. Intern. Med. (2021) (Epub ahead of print). 10.7326/M21-1757 PMC8407518 34461029

[15] Waissengrin B, Agbarya A, Safadi E, Padova H, Wolf I. Short-term safety of the BNT162b2 mRNA COVID-19 vaccine in patients with cancer treated with immune checkpoint inhibitors. Lancet Oncol. 22(5), 581–583 (2021). 33812495 10.1016/S1470-2045(21)00155-8 PMC8016402

[16] Chen YW, Tucker MD, Beckermann KE, Iams WT, Rini BI, Johnson DB. COVID-19 mRNA vaccines and immune-related adverse events in cancer patients treated with immune checkpoint inhibitors. Eur. J. Cancer 155, 291–293 (2021). 34400057 10.1016/j.ejca.2021.07.017 PMC8316066

[17] Denis M, Duruisseaux M, Brevet M, Dumontet C. How can immune checkpoint inhibitors cause hyperprogression in solid tumors? Front. Immunol. 11, 492 (2020). 32265935 10.3389/fimmu.2020.00492 PMC7098964

[18] Haeryfar SMM. MAIT cells in COVID-19: heroes, villains, or both? Crit. Rev. Immunol. 40(2), 173–184 (2020). 32749095 10.1615/CritRevImmunol.2020034943

[19] Flament H, Rouland M, Beaudoin L Outcome of SARS-CoV-2 infection is linked to MAIT cell activation and cytotoxicity. Nat. Immunol. 22(3), 322–335 (2021). 33531712 10.1038/s41590-021-00870-z

[20] Deschler S, Kager J, Erber J Mucosal-associated invariant T (MAIT) cells are highly activated and functionally impaired in COVID-19 patients. Viruses 13(2), 241 (2021). 33546489 10.3390/v13020241 PMC7913667

[21] Provine NM, Amini A, Garner LC MAIT cell activation augments adenovirus vector vaccine immunogenicity. Science 371(6528), 521–526 (2021). 33510029 10.1126/science.aax8819 PMC7610941

[22] Yatim N, Boussier J, Tetu P Immune checkpoint inhibitors increase T cell immunity during SARS-CoV-2 infection. Sci. Adv. 7(34), eabg4081 (2021). 34407944 10.1126/sciadv.abg4081 PMC8373136

[23] Gambichler T, Reuther J, Scheel CH, Susok L, Kern P, Becker JC. Cancer and immune checkpoint inhibitor treatment in the era of SARS-CoV-2 infection. Cancers (Basel) 12(11), 3383 (2021). 10.3390/cancers12113383 PMC7698088 33207589

[24] de Biasi S, Gibellini L, lo Tartaro D Circulating mucosal-associated invariant T cells identify patients responding to anti-PD-1 therapy. Nat. Commun. 12(1), 1669 (2021). 33723257 10.1038/s41467-021-21928-4 PMC7961017

[25] Kamar N, Abravanel F, Marion O, Couat C, Izopet J, del Bello A. Three doses of an mRNA covid-19 vaccine in solid-organ transplant recipients. N. Engl. J. Med. 385(7), 661–662 (2021). 34161700 10.1056/NEJMc2108861 PMC8262620

